# Anthracycline-Functionalized Dextran as a New Signal Multiplication Tagging Approach for Immunoassay

**DOI:** 10.3390/bios13030340

**Published:** 2023-03-03

**Authors:** Fatema Kaladari, Naoya Kishikawa, Ai Shimada, Mahmoud El-Maghrabey, Naotaka Kuroda

**Affiliations:** 1 Graduate School of Biomedical Sciences, Nagasaki University, 1-14 Bunkyo-machi, Nagasaki 852-8521, Japan; 2 School of Pharmaceutical Sciences, Nagasaki University, 1-14 Bunkyo-machi, Nagasaki 852-8521, Japan; 3 Department of Pharmaceutical Analytical Chemistry, Faculty of Pharmacy, Mansoura University, Mansoura 35516, Egypt

**Keywords:** immunoassay, signal multiplication, dextran, doxorubicin, chemiluminescence, colorimetry, enzyme-free tag, biotin

## Abstract

The most used kind of immunoassay is enzyme-linked immunosorbent assay (ELISA); however, enzymes suffer from steric effects, low stability, and high cost. Our research group has been developing quinone-linked immunosorbent assay (QuLISA) as a new promising approach for stable and cost-efficient immunoassay. However, the developed QuLISA suffered from low water-solubility of synthesized quinone labels and their moderate sensitivity. Herein, we developed a new approach for signal multiplication of QuLISA utilizing the water-soluble quinone anthracycline, doxorubicin, coupled with dextran for signal multiplication. A new compound, Biotin-DexDox, was prepared in which doxorubicin was assembled on oxidized dextran 40, and then it was biotinylated. The redox-cycle-based chemiluminescence and the colorimetric reaction of Biotin-DexDox were optimized and evaluated, and they showed very good sensitivity down to 0.25 and 0.23 nM, respectively. Then, Biotin-DexDox was employed for the detection of biotinylated antibodies utilizing avidin as a binder and a colorimetric assay of the formed complex through its contained doxorubicin redox reaction with NaBH_4_ and imidazolium salt yielding strong absorbance at 510 nm. The method could detect the plate-fixed antibody down to 0.55 nM. Hence, the application of Biotin-DexDox in QuLISA was successfully demonstrated and showed a significant improvement in its sensitivity and applicability to aqueous assays.

## 1. Introduction

Immunoassays that utilize antigen–antibody reactions are highly sensitive and highly selective for trace amounts, without the need for complicated pretreatment. Thus, they have been used in various fields, from basic research, such as toxicity analysis and clinical analysis [1,2,3,4]. Depending on the way the label differs in the antigen or antibody, an immunoassay can be classified into different types. Enzyme-linked immunosorbent assay (ELISA) uses an enzyme as a labeling tag that generates a signal [5]. In ELISA, a targeted antigen can be quantified by measuring the activity of the labeling enzyme after the formation of the enzyme-labeled immunocomplex (antigen–antibody). Compared with radioimmunoassay [6], which employs radioisotope labeling, ELISA is easier to handle, safer, and has excellent stability [7]. The signal generated in ELISA by the enzyme could be color development, fluorescence, or chemiluminescence (CL). A method wherein the enzyme is used to produce a CL signal is called a chemiluminescent enzyme immunoassay (CLEIA) [7,8]. CLEIA is widely used because of CL’s highly sensitive detection, simplicity, and wide dynamic range [9,10,11,12]. Generally, CLEIA utilizes label enzymes, such as horseradish peroxidase (HRP), that are able to produce a measurable signal in the form of CL [13]. However, enzymes are vulnerable to physical and chemical factors such as changes in temperature, pH, ionic strength, and solvent polarity, leading to denaturing and loss of their activity [14,15]. Moreover, the molecular size of the enzyme causes steric hindrance and non-specific adsorption, which results in high background noise [16,17,18].

Hence, non-enzymatic labels for detection are being and have been developed, such as beads [19] and metal nanoparticles [16,17,20,21,22,23,24,25]. Ye et al. used gold vesicles encapsulated with Pd-Ir nanoparticles (Pd-Ir NPs@GVs) as peroxidase mimics by raising the temperature. The gold vesicles captured by analytes liberate thousands of individual Pd-Ir NPs, which then act as the peroxidase mimics and generate an intense color signal by oxidation of TMB by hydrogen peroxide [26]. In addition, Li et al. used platinum Janus nanoparticles (Pt Janus NPs) as a replacement for the enzyme in Pt Janus NP-linked immunosorbent assays (Pt Janus NPs ELISA), where it works as a highly efficient peroxidase mimic that has a catalytic constant (Kcat) 140-fold higher than that of HRP [22]. Moreover, Yang et al. changed the enzyme with magnetic nanoparticles (MNPs) as a tag in CL immunoassays, and the CL signal was enhanced more than 20-fold by reacting MNPs with potassium hexacyanoferrate (II). Compared with traditional enzyme labels such as HRP, the used MNPs have exhibited excellent CL performance [25]. Others, like Higashi et al., have designed and used gold nanoparticles (AuNPs) instead of the enzyme, where AuNPs were used as a supporter for attaching antibodies and as electrochemiluminescence (ECL) probes [16]. AuNPs were able to produce reactive oxygen species (ROS) and react with luminol to improve the CL/ECL signal. Nevertheless, the developed methods still have some downsides, such as high cost, the vulnerability of nanoparticles, the complexity of synthesis, and their toxicity to the environment [27].

**Scheme 1:**
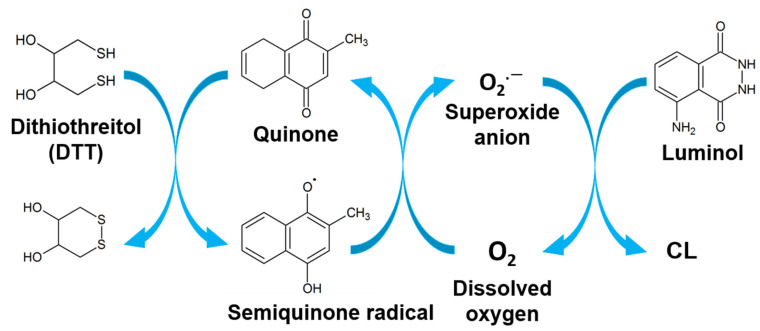
Mechanism of the redox cycle of quinone for CL detection.

**Scheme 2:**
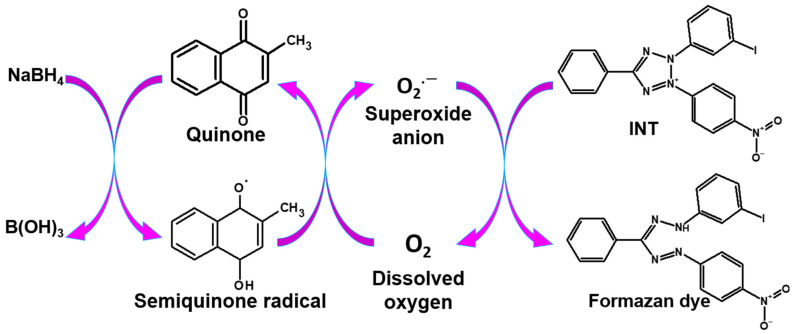
Quinone redox cycle mechanism for colorimetric detection.

Our research group has been concerned with and specialized in research on quinones and their use as labeling reagents, in addition to carrying out a thorough study of their redox cycle [28,29,30,31]. Our research team has lately focused on replacing the enzyme with quinones that were proven to be more stable; reproducible; and have a low molecular weight, which has less effect on the antigen–antibody reaction. The developed method is known as quinone-linked immunosorbent assay (QuLISA), which used the advantages of quinone for labeling in immunoassay [18,32,33,34,35,36]. As indicated in Scheme 1, the antibody-labeled quinone generates a superoxide anion radical when it reacts with a reductant such as dithiothreitol (DTT) via the redox cycle [32]. Additionally, quinone labels can react with sodium borohydride and INT, as shown in Scheme 2 for colorimetric assays [37]. The antibody can be determined by detecting this superoxide using luminol or tetrazolium dyes. As shown in Scheme 1 and Scheme 2, quinones react with a reductant (e.g., DTT or sodium borohydride), where it is reduced to semiquinone radicals; then, in the presence of dissolved oxygen, it produces a superoxide anion radical, which, upon the addition of luminol or INT, developed a CL or color signal, respectively.

There are other research groups that have used quinones in redox cycles, such as Xia et al. and Liu et al., where quinone was used as a booster in a colorimetric immunoassay by reduction of ferrozine into violet colored via tris(2-carboxyethyl)phosphine (TCEP) and to initiate an electrochemical-biochemical redox cycle to amplify enzyme signal by ferrocene carboxylic acid and TCEP, respectively. Nonetheless, these methods suffer from using an enzyme and non-selective adsorption [38,39].

However, the developed QuLISA so far faced one main problem, which is low to no solubility of quinone in water. Here, for the first time, using a water-soluble quinone called doxorubicin, we could synthesize a water-soluble quinone label that could be used as a new signal-generating tag in QuLISA. Moreover, additional signal amplification can be expected from labeling a single molecule of an antibody with many quinones bound to dextran. Lastly, by biotinylation, the polymer labeling of various biotinylated antibodies can be conducted through their binding of the water-soluble polymer by avidin, which will widen the applicability of the labeling reagent. As strong non-covalent avidin–biotin interactions have tremendously high affinity, fast on-rate, and high specificity, the bond between the two molecules is highly resistant to heat and acidic and alkaline pH.

**Figure 1:**
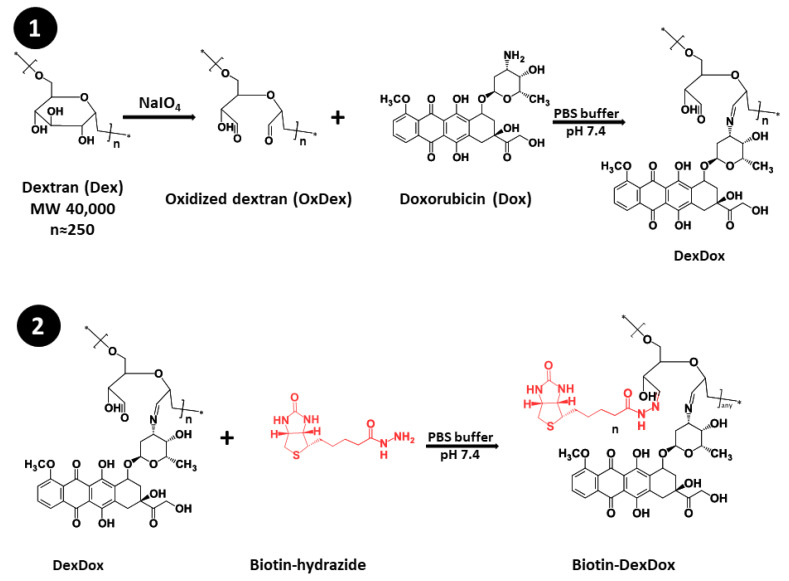
Preparation of Biotin-DexDox, including (**1**) the functionalization of the dextran with doxorubicin through its oxidation to oxidized, followed by amidation reaction with doxorubicin, and (**2**) biotinylation using biotin-hydrazide.

The novel approach for ultrasensitive detection based on signal amplification using a water-soluble quinone anthracycline, doxorubicin coupled with dextran, was invented for QuLISA, seeking water solubility. First, DexDox was prepared by oxidizing dextran 40, and then the water-soluble doxorubicin was assembled on the oxidized dextran (DexDox). The DexDox polymer was biotinylated, and the final polymer was called Biotin-DexDox (Figure 1). Biotin-DexDox was tested for its CL and colorimetric signal-generating capability and was employed for labeling biotinylated antibodies utilizing avidin.

## 2. Materials and Methods

### 2.1. Chemicals and Reagents

Luminol, dithiothreitol, bovine serum albumin (BSA), NaCl, NaHCO_3_, Na_2_CO_3_, H_2_SO_4_, HCl, CH_3_COONa, Na_2_HPO_4_, and CH_3_COOH were acquired from Nacalai Tesque (Kyoto, Japan). KCL, tween 20, hydrogen peroxide, *p*-iodonitrotetrazolium (INT), sodium borohydride, and KH_2_PO_4_ were sourced from Wako Pure Chemical Industries (Osaka, Japan). Anti-rabbit IgG-biotin conjugate and avidin were obtained from the following USA companies: ImmunoReagent Inc. (Burlington, NC, USA) and Calzyme Laboratories Inc. (San Luis, CA, USA), respectively. Biotin-hydrazide and NaIO_4_ were purchased from Sigma-Aldrich (St. Louis, MO, USA), while sodium hydroxide was from Merck (Darmstadt, Germany). Dextran 40 was obtained from TCI (Tokyo, Japan). Doxorubicin HCl was purchased from Funakoshi co (Tokyo, Japan). Double distilled water was used throughout the whole experiment, and it was produced from Yamato Autostill WG203 (Tokyo, Japan).

By combining sodium chloride, potassium dihydrogen phosphate, disodium phosphate, and potassium chloride, phosphate buffer saline (PBS, 100 mM pH 7.4) was prepared. A 50.0 mM carbonate-bicarbonate buffer with pH 9.6 was made from Na_2_CO_3_ and NaHCO_3_.

### 2.2. Instruments

The CL properties were evaluated using a Sirius luminometer (Berthold Technologies, Bad Wildbad, Germany). The microplate-based assays were performed in cell culture microplate, 96-well, PS, F-bottom from Chimney well, and white and transparent, CELLSTAR^®^, T, L.I.D., sterile, from Greiner Bio-One Co. Ltd., Tokyo, Japan. The microplate reader used was Spectra Max M5 and MaxL, from Molecular Devices (San Jose, CA, USA). The data processing software was Molecular Device, Softmax^®^ Pro 5 software. An F-71 pH meter (Horiba, Kyoto, Japan) was used for pH measurement. For spectrophotometry, a UV1800 UV/Vis spectrophotometer from Shimadzu (Kyoto, Japan) was utilized.

### 2.3. Preparing DexDox

First, DexDox was prepared by a similar method with slight modifications to Zhang et al. [40]; 250 mg of dextran 40 (MW 40,000 g/mol) was dissolved in 6 mL of water, and the solution was mixed with 0.25 M of sodium periodate (320 mg) incubated overnight at room temperature. Then, the solution was dialyzed against water using pur-A-Lzyer Maxi (MW 6000 Da), and 0.25 M of oxidized dextran was obtained. Next, 0.12 mL of 0.25 M oxidized dextran and 29.88 mL of 0.3 mmol of doxorubicin solution in PBS buffer (pH 7.4) were mixed and incubated overnight at 50 °C. After the incubation, the solution was dialyzed against 0.1 M acetate buffer (pH 4.2) to yield 4 µM of DexDox solution.

### 2.4. Synthesizing Biotin-DexDox

Biotin-DexDox was synthesized by mixing 1 mL of 4 µM of DexDox solution with 1 mL of 100 µM of biotin-hydrazide and incubated overnight at 50 °C. This was followed by dialysis against 0.1 M acetate buffer (pH 4.2). Then, 2 µM of Biotin-DexDox was obtained and stored in the fridge for further experiments.

### 2.5. Measuring the CL Intensity of Biotin-DexDox in the Luminometer

Different concentrations of Biotin-DexDox of 100 µL were added to test tubes, followed by 100 µL of 300 µM luminol in 0.08 M NaOH *aq*. Lastly, 100 µL of 100 µM DTT in water was added, and the CL intensity was measured for 10 min. 

### 2.6. Measurement of Biotin-DexDox in a Colorimetric Microplate Reader

An aliquot of NaBH_4_ (50 µL; 400.0 mM, in 60 mM NaOH) was added after adding INT (100 µL; 400.0 µM) and 50 µL of Biotin-DexDox from 1.0 to 200.0 nM to the microplate’s wells, respectively. After shaking for 5 s in the microplate reader, the microplate was inserted, and the absorbance was measured at 510 nm after 5 min [37].

### 2.7. Colorimetric Measurement of Microplate Immobilized Biotin-Labeled Antibody Using Biotin-DexDox

Standard solutions from 5 to 80 nM of biotin-labeled antibody dissolved in 0.05 M carbonate-bicarbonate buffer (pH 9.6) were prepared. Next, 100 μL of each standard was transferred to the microplate wells and incubated overnight at 4 °C. After that, 300 μL of 1% BSA was added and incubated at room temperature for 2 h for blocking free sites in the wells. Then, washing was carried out three times with PBS-T. Then, 100 μL of avidin (400 nM) aqueous solution was added and incubated for 1 h at room temperature. After the biotin-labeled antibody-avidin complex was formed, the wells were washed three times with PBS-T. Then, 100 μL of 640 nM of Biotin-DexDox solution was added and incubated at 37 °C for 2 h to label the biotinylated antibody–avidin complex. Lastly, the microplate was set in the microplate reader after the wells were washed three times, and INT and NaBH_4_ were added to each well; then, the color change was measured after 10 min at 510 nm.

## 3. Results and Discussion

### 3.1. Characterization of the Synthesized Product Biotin-DexDox

Dextran is a water-soluble polysaccharide that was used as the backbone of the synthesized functionalized polymer because it can be used after a chemical modification reaction, with promising properties and intriguing macromolecules possessing structural variety and functional versatility [41]. Among polysaccharides, dextran is widely used for medical and industrial applications [41].

The first step in the synthesis was to oxidize dextran, where hydroxyl groups were converted to aldehyde groups by sodium periodate, as shown in Figure 1. The percentage of sodium periodate to dextran is important because the water solubility of the dextran decreased with higher oxidation [40]. The second step is the biotinylation of DexDox via biotin hydrazide, where a hydrazone bond is formed between the biotin hydrazide and aldehyde groups in DexDox [42].

**Figure 2:**
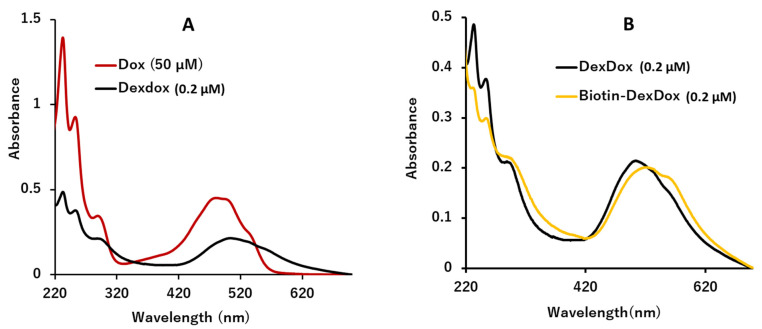
(**A**) UV spectra of 50 µM of doxorubicin and 0.2 µM DexDox and (**B**) UV spectra of 0.2 µM of DexDox and Biotin-DexDox.

Biotin-DexDox was characterized by colorimetric measurement of the dialyzed purified product at 495 nm [43]. The maximum absorbance peak of doxorubicin around 495 nm was clearly observed in biotin-DexDox, which clearly demonstrates the inclusion of doxorubicin in biotin-DexDox. Besides, in Figure 2B, there is a small shift in the absorbance of biotin-DexDox from that of DexDox, which indicates the inclusion of biotin.

Next, semi-quantitative characterization of Biotin-DexDox was carried out by comparing the absorbance of doxorubicin and DexDox [43,44]. From the results of the absorbance, as illustrated in Figure 2A, the relationship between 0.2 μM DexDox and 50 μM doxorubicin was found to be 0.46:1.0. The number of doxorubicin per one molecule was calculated using the following equation:

No of Dox = A_1_/A_2_ × Conc._2_/Conc._1_

where A_1_ and Conc._1_ are the absorbance of doxorubicin and its molar concentration, respectively, while A_2_ and Conc._2_ are the absorbance of Biotin-DexDox and its molar concentration, respectively. Applying the previously mentioned equation, it was deduced that there are approximately 115 doxorubicin molecules per one molecule of DexDox. This demonstrates the success of the developed signal multiplication approach through the confirmation of multiple functionalizations of dextran polymer with over 100 molecules of the anthracycline quinone, doxorubicin. Furthermore, UV measurements were performed to examine the stability before and after the biotinylation of DexDox, as shown in Figure 2B. It was confirmed that there was no problem or significant change in the stability of the polymer before and after its biotinylation.

In order to know how many biotin molecules are attached to any biotinylated compound, such as Biotin-DexDox, a reagent called 4′-hydroxyazobenzene-2-carboxylic acid (HABA) is used, which depends on the measurement of the absorbance of this reagent at 500 nm in the presence of avidin and avidin/biotinylated compound [36,45]. The presence of a biotinylated compound decreases the absorbance at 500 nm in a quantitative relationship to its molar content of biotin. This test was used by our research group in a previous publication [36]; however, when we tried to use the HABA test in the current method, it was not possible as the absorbance of doxorubicin is also at 495 nm; thus, it interferes strongly with the HABA assay at 500 nm. Consequently, it was very difficult for us to know how many biotin moieties are attached to Biotin-DexDox. Moreover, the labeling efficiency of Biotin-DexDox for immunoassay relies mainly on its doxorubicin (signaling molecule content), which was elucidated from the UV absorption curve, while biotin acts only as a binder, with no effect on labeling efficiency or sensitivity.

### 3.2. Evaluation and Optimization of the Redox-Cycle-Based CL Reaction of Biotin-DexDox

**Figure 3:**
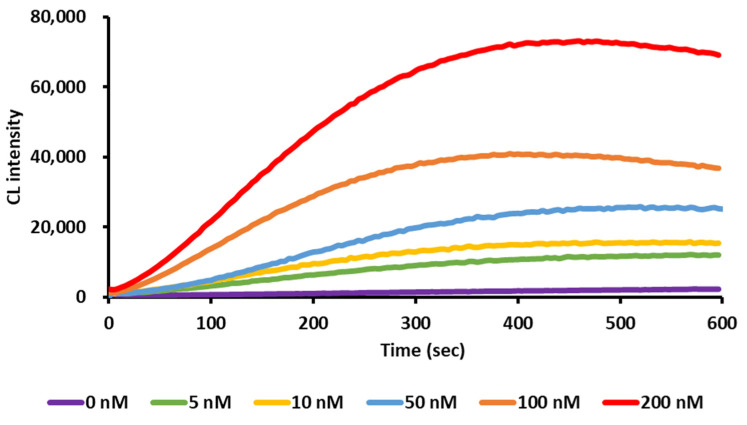
Time profile of the CL intensity of Biotin-DexDox.

Then, the CL behavior and intensity of several standard solutions of Biotin-DexDox upon its mixing with DTT and luminol were measured by the luminometer. Biotin-DexDox showed a glow-type CL that lasted for a long time, as shown in Figure 3. This happens as a result of the redox cycle of the quinone moiety (doxorubicin) present in the Biotin-DexDox. At first, DTT reacts with doxorubicin in Biotin-DexDox to produce a semiquinone radical. Then, the semiquinone radical reacts with dissolved oxygen and is recycled into quinone form, producing a superoxide anion radical. Then, the produced radicals reacted upon the addition of luminol, producing a three-amino phthalate excited state that, when returned to its ground state, forms strong CL. The produced CL was long-lasting as it was produced from a redox cycle reaction that is recycled from its own, as shown in Scheme 1.

**Figure 4:**
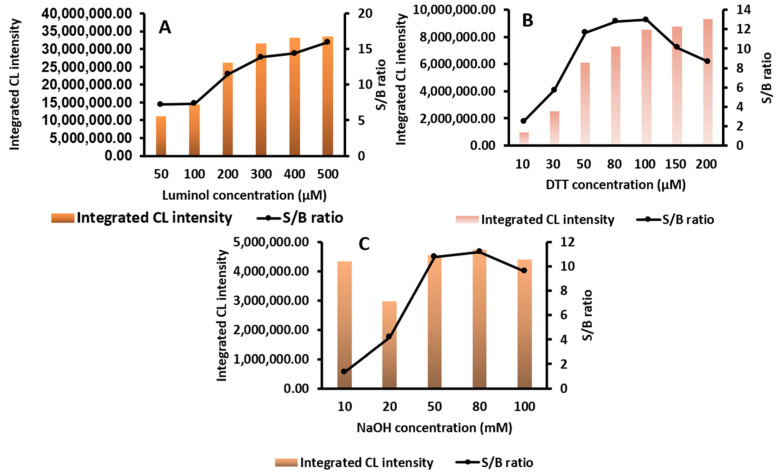
(**A**) Luminol concentration optimization in a luminometer displayed as integrated CL intensity and S/B ratio. (**B**) DTT concentration optimization in a luminometer displayed as integrated CL intensity and S/B ratio. (**C**) NaOH concentration optimization in a luminometer displayed as integrated CL intensity and S/B ratio.

**Figure 5:**
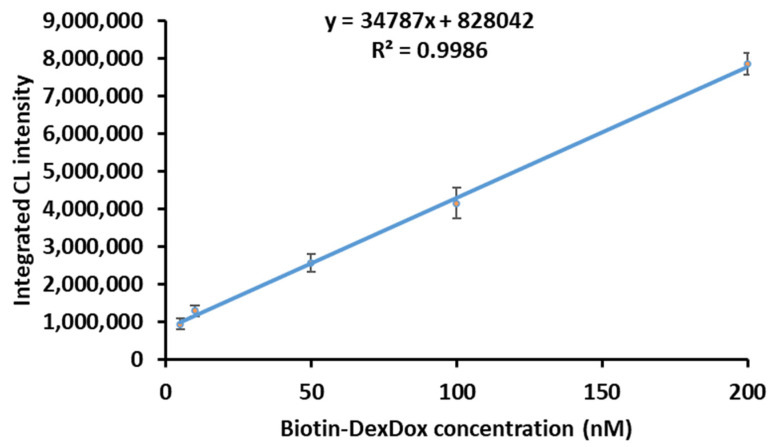
Calibration curve of Biotin-DexDox with the optimized CL conditions.

The redox cycle of quinone has been studied and used extensively in our laboratory, and different types of quinones have been screened [18,32,34,35,36]. However, in this article, we used a polymer consisting of biotin-dextran with 115 molecules of the quinone “doxorubicin” for the first time. Hence, the optimization of the conditions of the CL assay for this newly synthesized polymer is necessary to enhance the sensitivity and explore the full potential of the polymer. Consequently, the CL conditions were optimized, as shown in Figure 4A–C, to enhance the sensitivity for future applications. First, the luminol concentration was optimized in the range of 50–500 µM; an increase in the CL intensity was noticed from 50 µM to 300 µM; then, from 300 µM to 500 µM, it almost reached a plateau. The luminol concentration was chosen as 300 µM because it had an almost maximum and constant S/B ratio. The same was tested for DTT; from 10 to 200 µM, a dramatic increase in the CL intensity and the highest S/B ratio is observed at 100 µM, and then the S/B ratio starts to drop from 150 to 200 µM. Thus, 100 µM was chosen as the optimum concentration because it had the best S/B ratio. As shown in Figure 4C, the S/B ratio begins to increase from 10 to 80 mM and then slightly declines at 100 Mm. Thus, 80 mM of NaOH was chosen as the best concentration from the range of 10–100 mM. The linearity in enhanced conditions was conducted by preparing a Biotin-DexDox solution ranging from 5.0 to 200.0 nM (Figure 5) and with excellent linearity, with R^2^ = 0.998. The calibration curve followed the regression equation of y = 34787x + 828042, where y is the integrated CL intensity for 10 min and x is the concentration of Biotin-DexDox (nM). The limit of detection was calculated as 0.25 nM, described as 3σ/slope, where σ is the SD of the slope. This LOD is 31 and 6 times more sensitive than our previously developed CL-QuLISA using biotin-1,2-naphthoquinone (Biotin-NQ) [35] and CL-multi-QuLISA using Bio8mer-1,2-naphthoquinone (Bio8mer-NQ) [36], respectively, demonstrating the excellent sensitivity of our newly developed labeling agent Biotin-DexDox.

### 3.3. Evaluation and Optimization of the Redox-Cycle-Based Colorimetric Reaction of Biotin-DexDox

**Figure 6:**
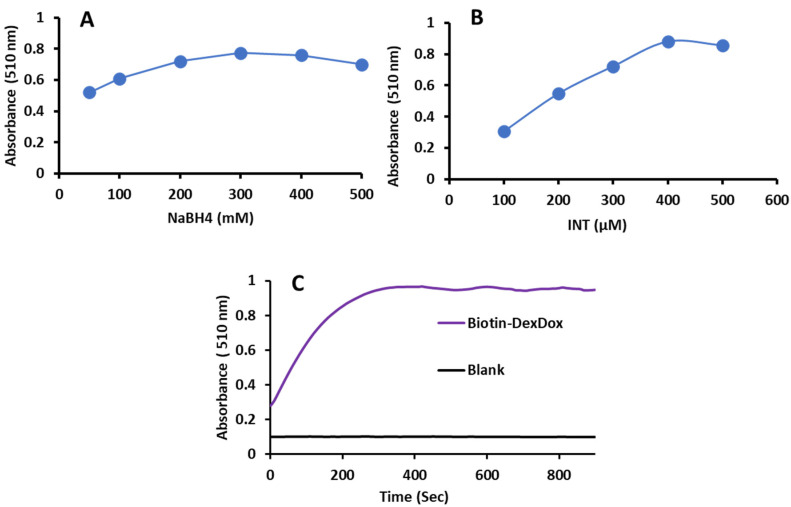
Effect of different parameters affecting the colorimetric reaction of Biotin-DexDox with NaBH_4_ and INT on the absorbance of the reaction products, including (**A**) NaBH_4_ concentration, (**B**) INT concentration, and (**C**) reaction time.

The Biotin-DexDox response was tested in the colorimetric assay, where a similar redox cycle system was utilized with sodium borohydride as a reductant, and INT as a colorimetric probe, where, when mixed in the presence of a superoxide anion radical, formazan dye with a maximum absorbance of 510 nm is developed (Scheme 2). Then, the colorimetric reaction conditions were optimized, as shown in Figure 6A–C, to enhance the sensitivity for future applications. First, NaBH_4_ concentration was optimized in the range of 50–500 mM; as illustrated in Figure 6A, the absorbance increases with the concentration and then reaches a plateau. Hence, 300 µM was picked because it yielded the highest absorbance. For INT, as shown in Figure 6B, a sharp increase in the absorbance is spotted when the concentration of INT increases from 100 to 500 µM, and 400 µM was chosen as the optimum concentration because it had the highest absorbance value. Lastly, the reaction time was studied for 15 min, and it was found that the colorimetric reaction reached plateau absorbance within 5 min.

**Figure 7:**
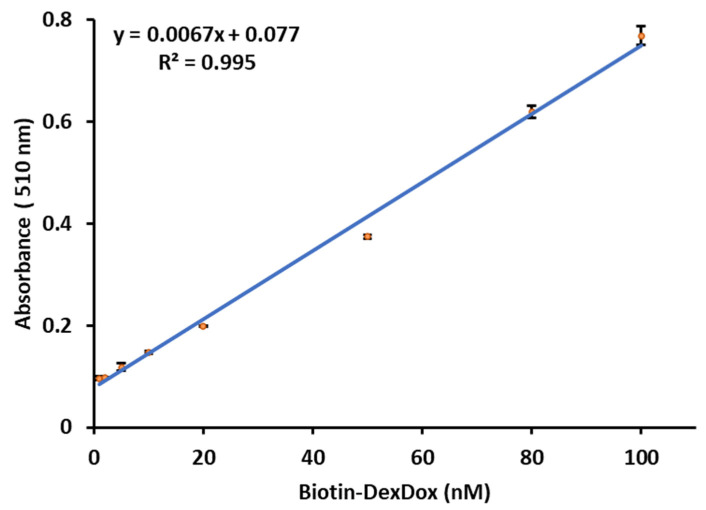
Calibration curve of Biotin-DexDox with the optimized condition for the colorimetric formazan dye method.

As shown in Figure 7, an excellent linear relationship was obtained between the absorbance and Biotin-DexDox concentration (nM), with R^2^ = 0.995. LOD was found to be 0.23 nM. This LOD is 213 and 28 times more sensitive than our previously developed QuLISA using Biotin-NQ [35] and multi-QuLISA using Bio8mer-NQ [36], respectively, demonstrating the superb sensitivity of the developed labeling agent. The greatly enhanced sensitivity in colorimetric mode obtained using doxorubicin derivative could be attributed to the high reactivity of doxorubicin with NaBH_4_ compared with that of NQ that was previously used by our research group. Moreover, the sensitivity of NQ in CL is eight times higher than that of the colorimetric method, while doxorubicin seems to have an equal reactivity towards DTT and NaBH_4_. This opens the door for future applications in both CL and colorimetric types of immunoassays.

### 3.4. Determination of Biotinylated Antibody via Biotin-DexDox Using Avidin and Redox-Cycle-Based Colorimetric Reaction

**Figure 8:**
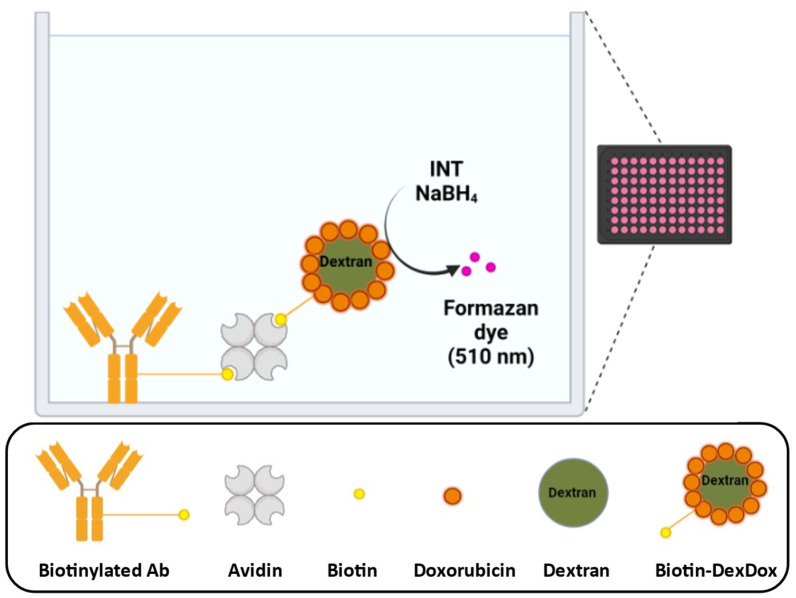
Simplified concept of using Biotin-DexDox for labeling immunoassays via the avidin–biotin system (made by Biorender.com, accessed on 28 January 2023).

The avidin–biotin system is known for its versatility and ease of preparation, and considering that the polymer will be used as a quinone label method for antibodies in immunoassays, biotin was introduced to DexDox to be used in the avidin–biotin system, as shown in Figure 8. Moreover, as labeling the antibody via the avidin–biotin system is easily prepared, it is superior to the method of directly labeling the antibody with quinone.

**Figure 9:**
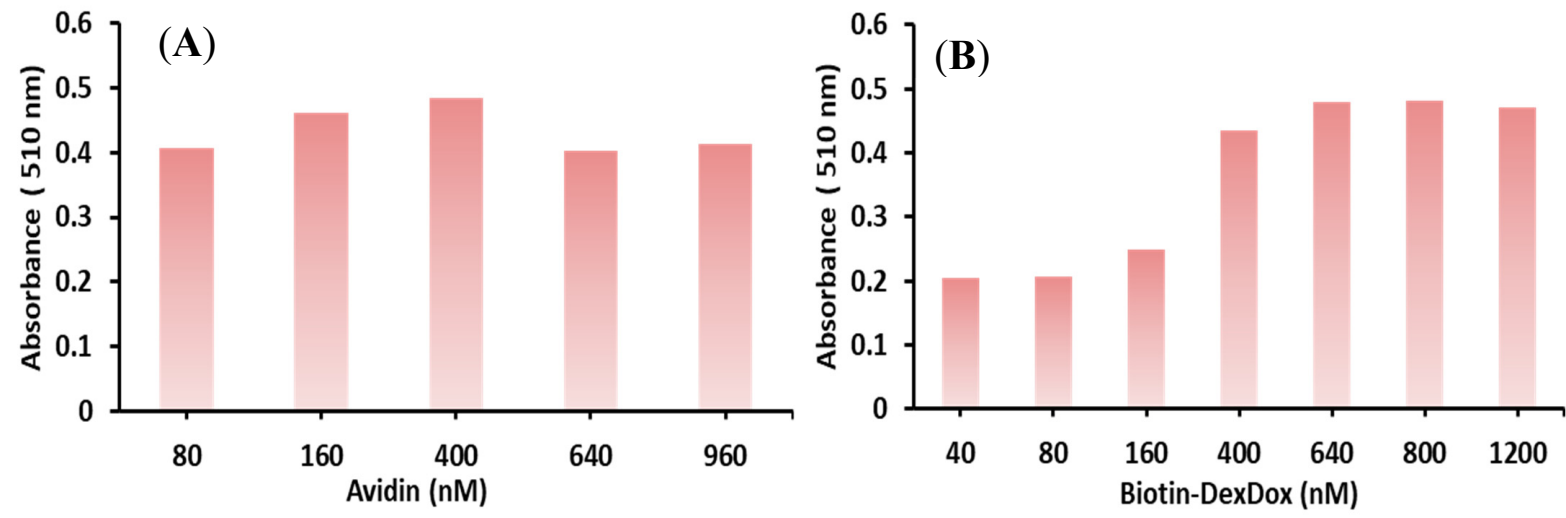
The effect of added (**A**) avidin concentration and (**B**) Biotin-DexDox on the determination of the well-fixed biotinylated antibody using the biotin–avidin interaction.

As colorimetric assays are inexpensive and have simple operations, they are widely utilized and available in most laboratories. Moreover, there is some limitation to the assays; for instance, CL intensity is not as precise as the absorbance. Furthermore, the newly developed labeling agent Biotin-DexDox has the unique property of having the same sensitivity in colorimetric, and CL measurement approaches. Additionally, colorimetric ELISA is the most available form; hence, the Biotin-DexDox response was tested using the colorimetric assay for the determination of biotinylated antibody. A proof-of-concept was conducted to investigate the ability of Biotin-DexDox to act as a signaling tag and maintain the redox cycle ability after binding to avidin. The concentration of biotinylated antibody concentration was evaluated using the avidin–biotin interaction. Firstly, a biotin-labeled IgG was fixed in the microplate well; then, avidin was added, followed by Biotin-DexDox. Subsequently, color development at 510 nm was generated by adding INT and NaBH_4_, and the color is proportional to the biotin-labeled antibody concentration. For the determination of the biotinylated antibody in optimum conditions, the avidin concentration was studied in the range of 1:1 to 12:1 of avidin–biotinylated antibody (avidin concentration in the range of 80–960 nM while maintaining the biotinylated antibody concentration at 80 nM). The optimum avidin concentration to achieve the highest sensitivity was avidin–biotinylated Ab in the ratio of 5:1 nM (400 nM avidin, Figure 9A). Then, the Biotin-DexDox concentration was studied in the range of 0.5:1 to 15:1 of Biotin-DexDox–biotinylated antibody (Biotin-DexDox concentration in the range of 40–1200 nM while maintaining the biotinylated antibody concentration at 80 nM). The optimum Biotin-DexDox concentration to achieve the highest sensitivity was Biotin-DexDox–biotinylated Ab in the ratio of 8:1 nM (640 nM avidin, Figure 9A).

**Figure 10:**
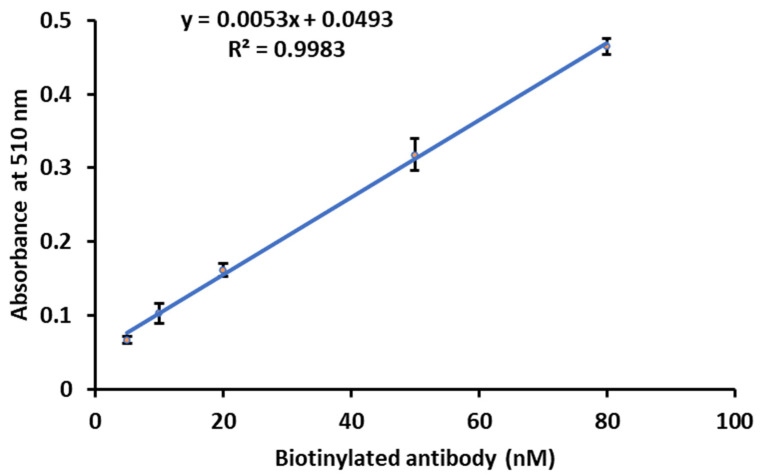
Calibration curve of the biotinylated antibody using avidin and Biotin-DexDox with the optimized conditions for the colorimetric INT assay.

A calibration curve was plotted between the concentration of the antibody on the y-axis and the absorbance on the x-axis. A straight-line calibration curve was obtained in the range of 5–80 nM biotinylated Ab, and LOD was found to be 0.55 nM (Figure 10). When the same experiment was carried out using biotin-HRP CL assay, the LOD was 63 nM [18,35], which means that Biotin-DexDox is 115 times more sensitive than the enzymatic labeling approach. This indicates that Biotin-DexDox can act as a signaling tag label for antibodies via the avidin–biotin interaction and can be used in immunoassays with excellent sensitivity.

## 4. Conclusions

Biotin-DexDox was designed to be a water-soluble quinone signal-generating tag for future application in immunoassay. Biotin-DexDox could be employed in both CL and colorimetric mode with excellent sensitivity down to 0.25 and 0.23 nM, respectively. The practicality of Biotin-DexDox as a possible label for antibodies used in the avidin–biotin system was examined, and LOD was found to be 0.55 nM for detecting biotinylated antibodies, which is 115 times more sensitive than the enzymatic labeling approach. On this basis, we could conclude that Biotin-DexDox is a water-soluble labeling agent for biotinylated antibodies that could be used for labeling multi-QuLISA with excellent sensitivity in both colorimetric and CL assays. Future applications for Biotin-DexDox are vast, owing to its high sensitivity, water solubility, and applicability in CL or colorimetric immunoassays, in any assay to label avidin where a color change or CL signal is needed for detection, and it is possible that this labeling polymer could even be used in fluorescence assays owing to the native fluorescence of doxorubicin.

## Data Availability

The data will be available upon reasonable request.
